# Imbalance of Mg Homeostasis as a Potential Biomarker in Colon Cancer

**DOI:** 10.3390/diagnostics11040727

**Published:** 2021-04-20

**Authors:** Davide Schiroli, Chiara Marraccini, Eleonora Zanetti, Moira Ragazzi, Alessandra Gianoncelli, Eleonora Quartieri, Elisa Gasparini, Stefano Iotti, Roberto Baricchi, Lucia Merolle

**Affiliations:** 1 Transfusion Medicine Unit, Azienda USL-IRCCS di Reggio Emilia, 42123 Reggio Emilia, Italy; Davide.schiroli@ausl.re.it (D.S.); Eleonora.Quartieri@ausl.re.it (E.Q.); Roberto.Baricchi@ausl.re.it (R.B.); Lucia.Merolle@ausl.re.it (L.M.); 2 Pathology Unit, Azienda USL-IRCCS di Reggio Emilia, 42123 Reggio Emilia, Italy; eleonora.zanetti@ausl.re.it (E.Z.); moira.ragazzi@ausl.re.it (M.R.); 3 Elettra—Sincrotrone Trieste, S.C.p.A., Basovizza, 34149 Trieste, Italy; alessandra.gianoncelli@elettra.eu; 4 Department of Medicine and Surgery, University of Parma, 43125 Parma, Italy; 5 Oncology Unit, Azienda USL-IRCCS di Reggio Emilia, 42123 Reggio Emilia, Italy; Elisa.Gasparini2@ausl.re.it; 6 Department of Pharmacy and Biotechnology, University of Bologna, 40127 Bologna, Italy; stefano.iotti@unibo.it; 7 National Institute of Biostructures and Biosystems, 00136 Rome, Italy

**Keywords:** colon cancer, magnesium, X-ray fluorescence microscopy, synchrotron light source, magnesium transporters, magnesium homeostasis

## Abstract

Background: Increasing evidences support a correlation between magnesium (Mg) homeostasis and colorectal cancer (CRC). Nevertheless, the role of Mg and its transporters as diagnostic markers in CRC is still a matter of debate. In this study we combined X-ray Fluorescence Microscopy and databases information to investigate the possible correlation between Mg imbalance and CRC. Methods: CRC tissue samples and their non-tumoural counterpart from four patients were collected and analysed for total Mg level and distribution by X-Ray Fluorescence Microscopy. We also reviewed the scientific literature and the main tissue expression databases to collect data on Mg transporters expression in CRC. Results: We found a significantly higher content of total Mg in CRC samples when compared to non-tumoural tissues. Mg distribution was also impaired in CRC. Conversely, we evidenced an uncertain correlation between Mg transporters expression and colon malignancies. Discussion: Although further studies are necessary to determine the correlation between different cancer types and stages, this is the first report proposing the measurement of Mg tissue localisation as a marker in CRC. This study represents thus a proof-of-concept that paves the way for the design of a larger prospective investigation of Mg in CRC.

## 1. Introduction

Colorectal cancer (CRC) represents the third most common malignant neoplasm with approximately 1.8 million new cases per year [1]. CRC carcinogenesis may arise from the accumulation of errors during DNA replication that mainly occur in repetitive DNA fragments and results in various genes mutations [2]. More than 90% of CRC are colorectal adenocarcinoma originating from epithelial cells [3]. Epigenetic changes are also known to cooperate with genetic alterations to drive the cancer phenotype [4]. Besides genetic and epigenetic mutations, lifestyle-associated risk factors are known to increase the incidence of this disease [5,6]: epidemiological studies suggest that smoking cessation, healthy diet and regular exercise can prevent the development of CRC [6]. 

Magnesium (Mg) has been proposed to have a preventive role in CRC: three independent meta-analyses inversely associated higher Mg intakes with a modest reduction in the risk of CRC [7,8,9], and a mouse model of induced CRC receiving Mg in drinking water was shown to suppresses colon carcinogenesis associated to inflammation [10]. Mg deficiency, due to either low Mg intake or excessive Mg waste, can be responsible of defects in DNA repairing mechanisms leading to genomic instability [11]. Hypomagnesaemia is frequent in patients with CRC [12,13] and serum Mg level detection was proposed as a biomarker of efficacy and outcome in patients with advanced CRC treated with cetuximab [14,15] and bevacizumab [16]. Hypomagnesaemia in cancer patients might be due to iatrogenic causes [17] or to the tumour itself, which may act as a Mg trap [18]. 

Mg is an essential element to the human body, acting as second messenger and being involved in over 600 enzymatic reactions, including energy metabolism, protein synthesis and cell replication [19,20]. Ionised Mg (Mg^2+^) is indeed the obligate counterion of all reactions that produce or consume nucleosides triphosphate, such as ATP, with second order enzymatic kinetics, meaning that small changes in free Mg^2+^ can elicit large effects in reaction rates [21]. Consequently, highly proliferating malignant cells should contain more Mg than resting ones due to their hyper-activation of energetic metabolism and ATP consumption [22].

Neoplastic cells can accumulate Mg by altering their extrusion mechanisms or by overexpressing Mg influx transporters. The activity of ion transporters in the gastrointestinal tract influences a variety of cellular processes, many of which overlap with the main cancer hallmarks. These proteins, indeed, play both oncogenic and tumour suppressive roles in the pathogenesis of malignant neoplasms [23]. The most accredited Mg influx channel involved in cancer is the transient receptor potential melastatin subfamily member 7 (TRPM7), which seems to be linked to tumour growth and metastasis on various types of cancer [24,25]. Other proteins of the TRP family such as TRPM6, which forms the Mg influx channel involved in intestinal and renal Mg absorption, are also emerging in this context [26].

Despite the premises, the real involvement of Mg and its transporters in cancer, and CRC in particular, remains controversial. Since only 1% of total Mg is found in blood [27], the clinical evaluation of its serum levels does not describe the actual content in tissues, and could even represent a deceptive information. Moreover, the mechanisms that regulate Mg cellular homeostasis and its correlation with Mg transporters have been mainly investigated in in vitro models and are still not completely elucidated [28,29]. Furthermore, it is still unclear which chemical fraction, whether free ionised magnesium (Mg^2+^) or bound to nucleotide or other macromolecules, may exert the most relevant biological effects [30]. The lack of effective methods able to detect Mg spatial distribution, as well as its tissue content and fluctuations to external stimuli, hampers the studies aimed at shedding light on the importance of Mg homeostasis in both pathological and normal conditions.

In this study, we assessed total Mg content and distribution through the X-Ray Fluorescence Microscopy (XRFM) analysis of CRC tissues [31]. We analysed cancer tissues from four different patients with same CRC diagnosis and staging. The data obtained were also integrated with literature and databases information on Mg transporters for which a possible involvement in CRC has been proposed. Although XRFM is not a practical method easily transferable to the routine clinical practice, these observations represent a proof-of-concept towards the quantification of Mg imbalance as a diagnostic marker in CRC.

## 2. Materials and Methods

### 2.1. Patients

This retrospective study was conducted at the AUSL-IRCCS di Reggio Emilia, Italy. We exploited formalin-fixed paraffin-embedded (FFPE) tumour and adjacent non-tumoural tissues, stored as histological blocks within the AUSL-IRCCS Research Biobank archive. In particular, we retrieved CRC tissue slices from four patients who underwent colon surgical resection. All patients were diagnosed with a T3N0 stage according to the criteria recommended by the World Health Organization Classification of Tumours [32]. This study was performed in accordance with the Declaration of Helsinki and approved by the Institutional Board Review (Reggio Emilia Ethical Committee Number 1197/2018/OSS/IRCCSRE).

### 2.2. Sample Preparation

Specimens were treated as previously described [31]. Briefly, the pathologist selected two histological blocks from each patient: one block almost entirely composed of cancer (CRC) and the other one taken from adjacent tissue as control (non-tumoural). Histological blocks were cut into 4-μm-thick slices, stained with haematoxylin and eosin (H&E) and inspected with a visible light microscope. On H&E slides the pathologist marked the most representative areas (i.e., the area with the higher percentage of neoplastic cells in the tumour tissue or the area with higher concentration of non-neoplastic cells in the non-tumoural tissue). Two additional consecutive 4-μm-thick slices were deposited on ultralene foils (3526, Ultralene^®^ pre-cut circles) for synchrotron XRFM analysis. 

### 2.3. Synchrotron Analysis and Microscope Setup

The measurements were carried out at the European twin X-ray microscopy station (TwinMic beamline) [33] of Elettra Synchrotron Trieste. The beamline is optimised for microscopy and Low Energy X-ray Fluorescence (LEXRF) and operates in the 400–2200 eV photon energy range. For our experiments, we used the Scanning Transmission X-ray Microscopy (STXM) operation mode of the beamline coupled with Low Energy X-Ray Fluorescence (LEXRF) analysis, in order to obtain information about tissue morphology and Mg content and distribution, respectively. 

### 2.4. Acquisition Protocol and Post-Processing

For the present experiment the samples were transversally scanned in the zone plate focus in steps of 1 μm at an incidence beam energy of 1470 eV in order to detect the K-line of Mg. STXM (absorption) images and fluorescence maps were acquired simultaneously. The exposure time was 10 ms per pixel for STXM images and 6 s per pixel for LEXRF. The total acquisition time per analysed area was in the range of 8–10 h (field of view of at least 70 × 70 μm; spatial resolution 1 μm). A total of four cases were analysed for both non-tumoural and for CRC tissue samples. X-ray Fluorescence intensity was measured by eight Si-drift detectors concentrically mounted at a 20 grazing angle with respect to the specimen plane, at a detector-to-specimen distance of 28 mm [34]. Zone plate, sample and detectors were in vacuum, thus avoiding any absorption and scattering by air.

The incident photon beam intensity was daily monitored through an empty area of the ultralene substrate by using a photodiode, in order to fairly compare the acquired maps. XRFM maps were deconvolved and analysed by using PyMCA software [35].

Mg content was evaluated by considering the total counts subtended by Mg K-line peak in the collected XRFM spectra. Besides investigating Mg distribution, by evaluating Mg XRFM emission point by point in the scanned area and producing Mg elemental maps, the total Mg counts in non-tumoural adjacent and CRC tissues were estimated by selecting the overall analysed areas, normalising the total counts by the size of area itself.

### 2.5. Literature and Database Analysis of Mg Transporters

Human Protein Atlas database was used to extract protein expression data of Mg transporter for both normal and cancer tissues (https://www.proteinatlas.org/, accessed date 26/01/2021). Genotype-Tissue Expression (GTEx) data were used to collect tissue-specific gene expression for normal tissues (https://gtexportal.org/home/, accessed date 26/01/2021).

Gene expressions of Mg transporters in cancer tissue were collected and analysed as follows: Gene Expression Omnibus (GEO) profiles of Mg transporters expression in colon cancer were browsed in GEO profiles (https://www.ncbi.nlm.nih.gov/geoprofiles/, accessed date 28/01/2021); dataset GDS2947 [36], GDS438:2 [37] and GDS2609 [38] were analysed; significant transporters are reported in Table 1. The Cancer Genome Atlas (TCGA) data were extracted from http://firebrowse.org/, (accessed date 28/01/2021); transporters found to be different in Colon Adenocarcinoma (COAD) compared to the controls and their relative fold change (FC) are reported in Table 1. Transporters reported in the Gene Expression Profile Interactive Analysis (GEPIA2) column of Table 1 are those found to be significantly different from the comparison between the TCGA cancer tissues and the GTEx normal tissues (http://gepia2.cancer-pku.cn/, accessed date 28/01/2021). Statistical significance was set at *p* < 0.05 (*t*-test) and FC > 1.1.

### 2.6. Statistical Analysis

Student’s *t* test was used to evaluate the differences of Mg levels between healthy and CRC tissue slices of the same patient. Statistical analysis was carried out on four non-tumoural adjacent and four CRC different regions. Data are presented as means ± SD of fluorescence counts normalised by pixels. Statistical analysis was performed by Graphpad Prim 6.0.

## 3. Results

**Figure 1:**
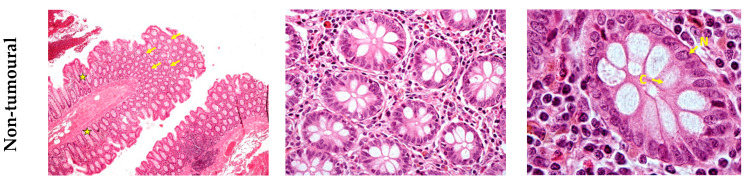

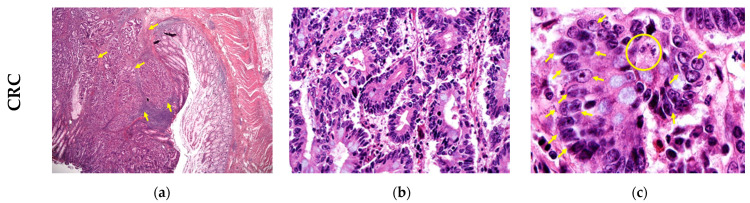
Histological inspection. Haematoxylin and eosin-stained representative regions of one out of four patients under study. Upper panel, non-tumoural adjacent colon shows the typical architecture with roundish (arrows) or elongated (stars) evenly spaced glandular structures lined by mucus secreting cells (**a**,**b**). In the lower panel, colorectal cancer presents crowded glands showing a cribriform or back-to-back growth pattern (arrows) (**a**,**b**). At higher magnification, normal gland shows a single layer of polarised cells with basal nuclei (N) and large cytoplasm (C) facing the lumen (**c**). Neoplastic glands show crowded enlarged nuclei (arrows) with prominent nucleolus and reduced cytoplasm. A mitotic figure is also present (see encircled nucleus) (**c**). The images were acquired at 40× (**a**) 400× (**b**) 1000× (**c**) magnification.

Tissue samples were stained with H&E and histologically inspected. Figure 1 reports the histological analysis of the adjacent non-tumoural (upper panel) and CRC (lower panel) tissues. Picture showing the non-tumoural tissue presents the typical features of the healthy colon epithelium characterised by glandular cells with circular or elliptic shapes. At higher magnification, normal gland shows a single layer of polarised cells with basal nuclei and large cytoplasm facing the lumen. On the other hand, CRC tissue presents crowded glands showing a cribriform or back-to-back growth pattern. Neoplastic glands show crowded enlarged nuclei (arrows) with prominent nucleolus and reduced cytoplasm. The area highlighted by a circle in Figure 1c lower panel represents a mitotic nucleus. 

**Figure 2:**
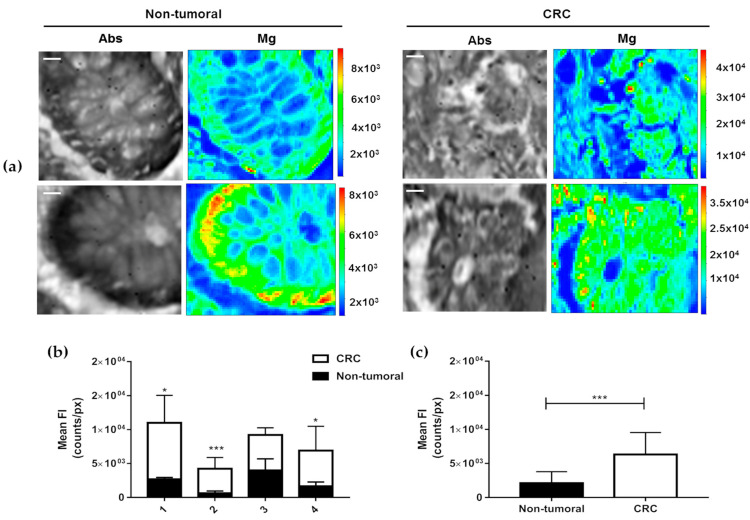
XRFM analysis. (**a**) Tissue morphology (Abs) and Mg content and distribution of non-tumoural colon epithelium (left panel) and CRC (right panel) tissues. The images shown are representative of the four patients under study; XRFM acquisition time= 6 s/pixel, Incident Energy= 1470 eV. Scale bars are 10 μm. Analysed areas: 78 × 78 μm^2^ and 78 × 60 μm^2^. (**b**) Mean fluorescence intensities of total Mg counts for each patient under study and (**c**) overall total Mg counts obtained considering all the Mg counts from each patient under study in non-tumoural adjacent and CRC tissues. Total counts were estimated by selecting the overall XRFM analysed areas and normalising the total counts by the size of area itself (pixels). Data are presented as mean ± SD. Non-tumoural (n = 21) and CRC (n = 15). The statistical significance was determined by Students’ *t*-test. * *p*-value < 0.05. *** *p* < 0.001.

The experiments performed at the TwinMic beamline (Elettra Synchrotron) show that Mg levels in cancer tissues are significantly higher than in the healthy ones. Representative results are reported in Figure 2: panel (a) depicts the absorption image (Abs) with the corresponding Mg XRF maps (Mg), panel (b) shows the total count of Mg per patient and panel (c) reports the overall Mg counts for all the analysed areas of the four patients of the study. For each patient, different areas of the same samples (CRC and non-tumoural) were analysed by XRFM: 21 non-tumoural and 15 CRC regions were evaluated providing, for each patient, the following average counts (reported as non-tumoural and CRC tissues, respectively): Patient 1, 2.65 × 10^3^ ± 2.91 × 10^2^ vs. 8.35 × 10^3^ ± 4.03 × 10^3^ (*p*-value = 0.015); Patient 2, 6.06 × 10^2^ ± 3.03 × 10^2^ vs. 3.65 × 10^3^ ± 1.65 × 10^2^ (*p*-value = 0.0003). Patient 3, 3.97 × 10^3^ ± 1.73 × 10^3^ vs. 5.25 × 10^3^ ± 1.06 × 10^3^ (*p*-value = 0.3746); Patient 4 1.64 × 10^3^ ± 6.32 × 10^2^ vs. 5.27 × 10^3^ ± 3.59 × 10^3^ (*p*-value = 0.0474).

From the absorption images, which delineates the sample morphology, it appears that colon mucous glands are roundly shaped and well defined for control samples, where lumen, cytoplasm and nuclei are clearly visible. On the other hand, in CRC samples the mucous glands appear almost smashed, fallen apart and, therefore, not easily recognisable; they seem almost a continuum with the rest of the tissue.

Moreover, in control specimens Mg is clearly localised on the nuclei located on the border of the glands, while on CRC ones it is more uniformly distributed across the gland area.

Mg transporters known to be expressed in the colon [39] were screened for database and literature analysis (Table 1).

Most of the transporters were found both at the RNA (GTEx) and protein (Human Protein Atlas) level, while there is no validated antibody reported in Human Protein Atlas able to detect TRPM6. The latter, however, was previously observed to be highly expressed in colon [40,41]. The analysis of the expression levels of these transporters in colon malignant and pre-malignant conditions in comparison with healthy controls has often brought to uncertain results. TRPM6 and TRPM7 were recently reported to be overexpressed at protein levels in both CRC and pre-cancerous conditions, such as intestine bowel disease [41,42]. On the other hand, based on both the TCGA and GEO profiles analysis, mRNA was found to be down-regulated for TRPM7 and unaltered for TRPM6. Some data support the hypothesis that also Mitochondrial Inner Membrane Magnesium Transporter (MRS2), Magnesium Transporter Protein 1 (MAGT1), Solute carrier family 41 member 1 (SLC41A1), Metal Transporters CNNM1 and CNNM4 may be dysregulated in colon cancer [36,38]; however, more studies are necessary to confirm these observations. Conversely, to our knowledge there is no study or dataset reporting a significant correlation between CNNM3 expression and colorectal cancer.

## 4. Discussion

Mg plays a pivotal role in the human body; however, its involvement in cancer has long been debated, with many studies that produced conflicting results [46,47,48]. This discrepancy may in part be attributed to the lack of adequate analytical techniques able to monitor tissue distribution, fluxes and interactions between trace elements with the necessary sensitivity [49,50]. Indeed, currently available techniques to assess Mg lack of sensitivity and are not able to effectively investigate ion fluxes in response to a number of stimuli, thus hampering the studies on Mg imbalance in CRC and other pathologies. Besides the doubts concerning the role of Mg transporters and the mechanisms that regulate its homeostasis, it is also still unclear whether the free or bound Mg form may exert the most significant biological effects [30].

Despite not being easily transferrable to clinical practice, XRFM has recently been unveiled as a powerful tool for imaging tissues at micron and sub-micron scales, as demonstrated by our previous data on thyroid cancer [31]. Being label-free, it provides direct qualitative and quantitative elemental spatial distribution with high resolution, high penetration power and sensitivity.

In this study, we exploited XRFM to investigate Mg content in CRC tissues, finding a higher overall Mg content than in their non-tumoural counterpart with a statistical significance difference. These results are in agreement with our previous in vitro data on colon cancer cell lines, where total Mg was found to be directly correlated to cell proliferation and drug-response [28,51]. To our knowledge, this is the first attempt to quantify and map total Mg at cellular level in human CRC tissues. In non-tumoural tissue samples, the highest Mg content is found in the nuclear and perinuclear region, where mitochondria are localised. Indeed, mitochondria are known to be the intracellular Mg store [29,52]. Therefore, the Mg distribution found reflects the normal cell physiology. On the other hand, in CRC samples the extreme tissue disorganisation leads to a disordered Mg distribution, whose functional consequences need to be investigated.

Of note, XRFM is a non-destructive analytical method that, in the last decade, has been extensively employed to investigate the distribution and content of Mg [53,54,55] and other chemical elements in tissue and cells [56,57,58,59,60]. Clear-cut evidence has been provided that the inherent limitation of single cell analysis approach is largely manageable as far as the assessment of intracellular Mg concentration [61].

Since the increase of total Mg observed in cancer tissues may be due to enhanced influx and/or increased Mg-binding mediated by ion transporters, we dug through literature and databases for the expression of Mg transporters and their possible involvement in CRC insurgence and progression. Intestine represents the main absorption site of Mg, which is next stored in bones and excreted by kidneys. About one-third (100/300 mg) of the daily Mg intake is mainly absorbed in the small intestine via paracellular absorption and through TRPM6 and TRPM7 active transport in the cecum and colon [62,63]. Our literature and database analysis was performed integrating and expanding the information recently collected by Auwercx and colleagues [39]. In our work we focused specifically on CRC, introducing further comparisons that consider TCGA normal samples and GEO profiles. The evidences collected in Table 1 show that, despite most of Mg transporters found to be dysregulated in colon malignancies, a correlation between their expression and pathological conditions remains largely uncertain. TRPM6, for instance, was reported to be overexpressed at protein level in both CRC and in pre-cancerous lesions [41], while genetic expression [43] and mRNA-based studies seem to suggest the opposite (Table 1). TRPM7, whose protein was found to be overexpressed in CRC [41,42] and adenocarcinoma tissues [41], was instead unaltered at the mRNA level. Actually, many scientific pieces of paper claim a role of TRPM7 in CRC and other cancers [22,24,25,41,42,64]. Our literature screening, however, indicates that the real involvement of TRPM6 and TRPM7 channels in CRC deserves in-depth evaluation. In particular, future studies on TRPM7 should evaluate not only protein expression, but also its genetic mutations and polymorphisms, that have already been found in various types of carcinoma [25].

Taken together, these observations suggest that the quantification of tissue Mg may potentially become a much more reliable approach towards the identification of a novel marker for CRC.

Indeed, convincing evidence demonstrates the peculiarity of the cellular magnesium ion homeostasis: as a consequence of different hormonal and non-hormonal stimuli, substantial amounts of free Mg^2+^ have been shown to flow across the cell membrane in both directions, resulting in substantial changes in the amount of cation present in human plasma serum [65] and in signal transduction pathways activation [20]. However, there is reasonable scepticism about Mg as second messenger, mainly because of its abundance within the cell and the relative difficulty to discriminate its contribution to cell signalling [21]. Indeed, Mg trafficking results in small changes of free Mg^2+^ intracellular concentration, while large variations of its total content have been found in subcellular organelles and tissues [66]. Therefore, the use of specific and sensitive tools capable of detecting and quantifying the total magnesium content in cells and tissues represents an essential approach for biological and biomedical research in this field [61,67].

These aspects should be investigated considering that more than 600 enzymes require Mg as a co-factor. Among them, the mammalian target of rapamycin (mTOR) is responsible of an enzymatic cascade that regulates cell proliferation, autophagy, and apoptosis by participating in multiple signalling pathways, and has also been associated with cancer. Under normal circumstances, mTOR is a major regulator of cell growth and division. In tumour cells, however, abnormally activated mTOR sends signals that encourage tumour cells growth, metastasis, and invasion [68]. In particular, the PI3K/Akt/mTOR signalling pathway has been found to control the proliferation and survival of colon cancer stem cells, and seems to be linked to colon cancer recurrence and metastasis [69]. Recent in vitro studies demonstrated the correlation between Mg fluxes and activation of mTOR signalling pathway [70].

Since Mg fluxes are tightly regulated by several transporters, we believe it is mandatory to deeper investigate these aspects in cancer patients, eventually combining Mg transporters expression and polymorphisms to Mg levels: these data might be essential for identifying a diagnostic tool based on the Mg homeostasis in CRC. Immunohistochemistry has already been used to characterise Mg channelsTRPM6 and TRPM7 in CRC and non-tumoural tissues [41]: it would be useful to exploit this technique in future studies.

Although the XRFM technique is not easily affordable and unlikely applicable in the clinical routine, we believe that, with the advent of more powerful laboratory X-ray sources and detectors, this method could be transferred to future clinical laboratories. Finally, being a retrospective study on already deposited biobank tissues, we could not match our data with the serum Mg levels of the selected patient. Indeed, serum Mg is not routinely analysed in the clinical practice, despite its imbalance being linked to several pathological conditions [62,63,71]. 

Future large-scale prospective studies will have to take into consideration all these technical limitations. Moreover, we are aware that this study, albeit being an innovative proof-of-concept, lacks of the adequate sample size to ensure power and robustness to our observations.

## 5. Conclusions

The results presented herein provide evidences of a direct correlation between total Mg content and distribution in tissue and CRC. Our observations highlight the importance of developing a more comprehensive analysis of Mg levels in CRC. Further studies may also take into consideration the intracellular levels of Mg together with the expression of proteins involved in Mg metabolism, the genomic differences and the concentrations of other ions, in order to better stratify colon cancer patients. Moreover, the correlation between Mg intake and its concentration in cells (of colon and other tissues) and body fluids (serum and urine) is a matter of debate that still deserves to be investigated [62,72], as this ion might still become an effective diagnostic tool. 

## Figures and Tables

**Table 1:** Gene and protein expression profiles of Mg channels and transporters in humans.

x	Expression in Normal Tissue		Expression in Cancer Tissue				
	GTEx	Human Protein Atlas	Human Protein Atlas	TCGA	GEPIA2	GEO Profiles	Literature
TRPM6	Mainly expressed in colon, Brain and Testis	Not detected	Not detected	Down-regulated in COAD (FC 30)		Down-regulated in CRC (FC 1.25) [37] Down-regulated in CA (FC 15) [36]	Higher in CRC (IHC) [41] Down-regulated in CRC [43]
TRPM7	Ubiquitous, expressed in colon	Moderate in colon, only glandular cells	No expression in 11 CRC patients				Higher in CRC (IHC) [41] Higher in CRC (IF) [42]
MRS2	Ubiquitous, expressed in colon	High in colon glandular cells and moderate in endothelial cells	Strong and moderate expression in 11/12 CRC patients			Down-regulated in early-onset CRC (FC 1.3) [38] Up-regulated in CA (FC 1.3) [36]	
MAGT1	Ubiquitous, expressed in colon	High in colon glandular cells and moderate endothelial cells	Moderate expression in 10/12 CRC patients		Up-regulated in COAD (FC 2.5)		Up-regulated in CRC (mRNA) [44]
SLC41A1	Ubiquitous, expressed in colon	High in colon glandular cells and moderate in endothelial cells	Strong and moderate expression in 12/12 CRC patients			Up-regulated in early-onset CRC (FC 2.15) [38] Up-regulated in CA (FC 1.21) [36]	
CNNM1	Low expression in colon	Moderate expression in colon, in both glandular and endothelial cells	Low and moderate expression in 7/12 CRC patients			Down-regulated in CA (FC 1.33) [36]	
CNNM3	Ubiquitous, expressed in colon	High expression in colon glandular cells, moderate in Colon endothelial cells	Strong and moderate expression in 12/12 CRC patients				
CNNM4	Ubiquitous, expressed in colon	High expression in colon, only in glandular cells	Strong and moderate expression in 10/12 CRC patients	Down-regulated in COAD (FC 3.4)	Up-regulated in COAD (FC 3)	Down-regulated in CRC (FC 1.18) [37] Down-regulated in early-onset CRC (FC 1.94) [38] Down-regulated in CA (FC 2.6) [36]	Lower in colon cancer-derived Metastases (IHC) [45]

Abbreviations: FC: Fold Change, CA: Colorectal Adenoma, COAD: Colon Adenocarcinoma, CRC: Colorectal Cancer, IHC: Immunohistochemistry, IF: Immunofluorescence.

## Data Availability

All datasets analysed are public and available and they were extracted from: Human protein atlas (https://www.proteinatlas.org/, accessed date 26/01/2021), Genome Tissue Expression (GTEX) project (http://www.GTEXportal.org/, accessed date 26/01/2021), TCGA Research Network (http://cancergenome.nih.gov/, accessed date 26/01/2021, data were extracted from http://firebrowse.org/, accessed date 26/01/2021), and Gene Expression Omnibus (GEO) database (https://www.ncbi.nlm.nih.gov/geoprofiles/, accessed date 26/01/2021) or using GEPIA2 (http://gepia2.cancer-pku.cn/, accessed date 26/01/2021).
